# Moving Accelerometers to the Tip: Monitoring of Wind Turbine Blade Bending Using 3D Accelerometers and Model-Based Bending Shapes

**DOI:** 10.3390/s20185337

**Published:** 2020-09-17

**Authors:** Theresa Loss, Alexander Bergmann

**Affiliations:** Institute of Electrical Measurement and Sensor Systems, Graz University of Technology, Inffeldgasse 33/I, 8010 Graz, Austria; alexander.bergmann@tugraz.at

**Keywords:** blade bending, accelerometers, condition monitoring, wind energy

## Abstract

Increasing the length of wind turbine blades for maximum energy capture leads to larger loads and forces acting on the blades. In particular, alternate bending due to gravity or nonuniform wind profiles leads to increased loads and imminent fatigue. Therefore, blade monitoring in operation is needed to optimise turbine settings and, consequently, to reduce alternate bending. In our approach, an acceleration model was used to analyse periodically occurring deviations from uniform bending. By using hierarchical clustering, significant bending patterns could be extracted and patterns were analysed with regard to reference data. In a simulation of alternate bending effects, various effects were successfully represented by different bending patterns. A real data experiment with accelerometers mounted at the blade tip of turbine blades demonstrated a clear relation between the rotation frequency and the resulting bending patterns. Additionally, the markedness of bending shapes could be used to assess the amount of alternate bending of the blade in both simulations and experiment.s The results demonstrate that model-based bending shapes provide a strong indication for alternate bending and, consequently, can be used to optimise turbine settings.

## 1. Introduction

With a newly installed wind energy capacity of 60.4 GW worldwide, installations increased by 19% in comparison to 2018 and finally started to contribute to the global demand to reduce carbon emissions [1]. Maximising the energy capture per turbine results in fewer turbines per farm, thereby leading to a reduction in the levelised costs of energy. This aspect even comes into effect considering the higher costs of the components of larger turbines [2].

During the last 20 years, the energy capture per turbine could significantly be enhanced by increasing the height and diameter of turbines. For instance, the average rated newly installed capacity in the United States of 2.43 MW at an average rotor diameter of 115.6 m in 2018 corresponds to an increase of 239% in capacity and of 41% in blade length in comparison to the year 1998 [3]. Moreover, the largest prototype turbine in the world started to operate with a rotor diameter of 220 m in 2019 [4].

Intense research is being conducted on blade design since the relative mass and volume of the blade per meter needs to be reduced when designing longer and more efficient blades. Longer blades also experience higher aerodynamic and gravitational loads on the structure itself as well as on pitch bearings and drive trains [2,5].

### 1.1. Relevance

While aerodynamic simulations help blade engineers to design blade geometries and material composites, blade monitoring in operation is particularly important to assess blade behaviour. First, continuous monitoring allows for detecting damage and irregular behaviour of the blades at an early stage, thereby reducing consequential damage. This helps to reduce maintenance and repair costs and shortens standstill times in comparison to scheduled visual inspection [6]. Second, safety of technicians on site as well as of individuals is increased. Damage types such as breaking of components of the blade, partial blade breaks, and even loss of a blade can be prevented if detected at an early stage [7]. Third, monitoring in operation can be used to optimise turbine settings and to examine blade behaviour. Consequently, continuous monitoring helps to adjust simulations and comes full circle.

**Figure 1:**
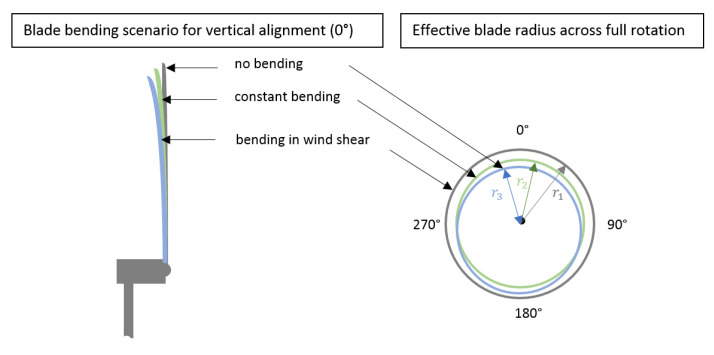
Sketch of blade bending for (i) no bending, (ii) constant bending, and (iii) bending due to wind shear: visualisation of blade bending (**left**) and effective blade radius across rotation (**right**).

In our approach, we focus on continuous monitoring regarding bending of the blade. During its typical lifetime of 20 years, a blade experiences more than 28 million rotations based on 4000 h of operations per year at an average rotational speed of 0.1 Hz. Ideally, blades experience quasi-static wind loads and constant bending. However, blades are also affected by dynamic wind effects such as periodic wind effects, i.e., vertical and horizontal wind shear (yaw), as well as aperiodic wind effects, i.e., turbulences, which lead to periodic and aperiodic bending, respectively [8]. Additionally, gravity leads to alternate bending of blades, especially at low wind speeds when stiffening effects are low. Figure 1 visualises alternate bending in the case of wind shear, i.e., wind speed decreasing from top to bottom relative to the turbine.

Deflection and the resulting stress during alternate bending increase with blade length and therefore gain importance in current blade trends. Over the lifetime of a turbine, alternate bending leads to increased loads and resulting fatigue. Therefore, online monitoring of blade bending is needed to assess the extent of alternate bending and to optimise turbine settings.

### 1.2. Related Literature

Simulating periodic wind effects and the resulting behaviour of the blades helps to understand the forces acting on the blades. Kragh and Hansen [9] studied the potential to use yaw misalignment for reducing blade loads in wind shear conditions by simulating a 5 MW reference turbine. Their results showed that steady-state load variations could be reduced by adapting yaw misalignment depending on the turbulence level. Dai et al. [10] studied aeroelastic modelling by comparing aerodynamic loads and deflections for two different solving algorithms. While maximum deflection in yaw conditions occurred at about 90 and 270 azimuth angle, the authors could also show that results varied for different solving algorithms. Additionally, Ke et al. [11] investigated wind-induced fatigue of large turbines in periodic wind effects. Furthermore, Liew et al. [12] studied individual pitch control (ICP) to prevent alternate bending by guiding blades along a preset trajectory but did not consider feasible sensor solutions for measuring tip deflection yet.

Simulations offer a cheap and fast way to evaluate blade bending but require an estimate of material properties and wind effects. A mismatch between simulated properties and actual behaviour of the blade can only be detected by performing measurements in operation. For example, White et al. [13] reported a mismatch of simulated and experimentally measured eigenfrequencies. Therefore, simulations need to be complemented by continuous measurements during operation.

Mounting sensors on the blade is promising for determining blade position and bending in operation. Accelerometers have been used to determine blade eigenfrequencies for damage detection in various structural health-monitoring approaches [14,15]. Also, White et al. [16] studied tip deflection by using an array of accelerometers on a 9 m smart blade and presented promising results in the first deflection tests. However, sensors were placed on the inside of the blade, where mounting positions are limited due to support structures. In contrast, Loss et al. [17] mounted a triaxial accelerometer on the outside of a blade at 90% of the blade length. Features regarding nonuniform movement of the blade were extracted; however, an analysis on bending shapes across the rotational movement of the blade has not been conducted yet. Additionally, Fu et al. [18] installed triaxial gyroscopes at 20% of the blade length and trained an artificial neural network to detect tip clearance. Although gyroscopes are promising in blade monitoring, stability and robustness of the purely on artificial intelligence based method have not been evaluated.

Besides mounting sensors on the rotor blade itself, remote sensing has also been studied for monitoring blade bending. Zhang et al. [19] embedded antennas into the blade tip as well as on the outside of the blade root for ultra-wideband (UWB) sensing. Therefore, deflection could be estimated with a maximum deviation of 0.13 m when performing measurements on a test rig. Additionally, Moll et al. [20] used a radar-based system for detecting artificially introduced damage in rotor blades. However, monitoring was restricted to the measurement range of the radar system in front of the turbine tower. Finally, Grosse-Schwiep et al. [21] tested laser scanning for detecting blade shapes. However, Yuan [22] reported drawbacks such as a trade-off between sensitivity and costs as well as safety hazards of high-power lasers in the related field of aerospace structures.

### 1.3. Approach and Objective

In our approach, we measure acceleration at the blade tip to detect deviations from the ideal uniform rotation. By using a model solely based on measured acceleration, our method is independent of material properties and exact blade geometry, which are often not available from the manufacturer. The two main objectives of our method are the optimisation of turbine settings for reducing alternate bending as well as the detection of deviating bending behaviour due to damage of components. By mounting energy self-sufficient sensors on the outside of the blades, our solution allows for flexible mounting positions and can also be used for retrofitting existing turbines.

This paper is structured as follows: Section 2 presents our method including the model of acceleration measurements (Section 2.1), the calculation of bending shapes representing characteristic blade bending (Section 2.2), and the pattern recognition task (Section 2.3). A simulation of periodic wind effects and the resulting bending shapes is included in Section 3. Section 4 presents a real data experiment using triaxial accelerometers mounted at a minimum of 80% blade length on two different test turbines. Finally, the results are discussed in Section 5 and conclusions are drawn in Section 6.

## 2. Method

**Figure 2:**
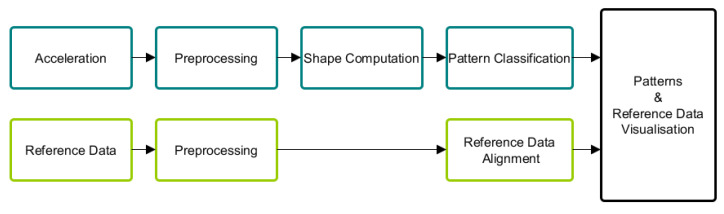
Overview of data processing.

The process for creating model-based bending shapes from acceleration measurements consists of six parts (see Figure 2): First, a model for simulating acceleration measured by a sensor at any position and orientation on the blade was developed (Section 2.1). Second, preprocessing was applied to filter steady-states of the turbine, which will be described in further detail in context with real data evaluation (Section 4.2). Then, bending shapes were computed, which indicate the deviation from measured acceleration to the ideal uniform rotational movement (Section 2.2). Bending shapes were then grouped to distinct patterns and used to create a morphing circle, which arranged bending shapes with regard to their similarity (Section 2.3).

### 2.1. Model of Acceleration Measurements

**Figure 3:**
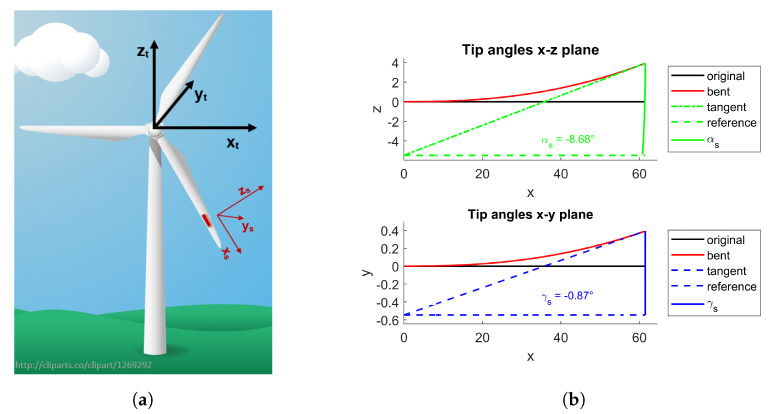
(**a**) Coordinate systems of the turbine and sensor and (**b**) the resulting change in orientation of the sensor due to blade bending, adapted from [17].

First, the coordinate system of the acceleration model is specified in the following. The turbine coordinate system (referred to as axes $x_{t}$, $y_{t}$, and $z_{t}$ in the following) was aligned to the centre of the turbine hub, as shown in Figure 3a, with the rotation angles being defined as the following:

- $\alpha_{t}$: rotation around the $y_{t}$-axis due to rotational movement of the blade.
- $\beta_{t}$: pitch angle of the blade around the $x_{t}$-axis.
- $\gamma_{t}$: orientation of the blade around the $z_{t}$-axis, e.g., due to bending.

The measurement axes of the accelerometer are specified as $x_{s}$, $y_{s}$, and $z_{s}$. The orientation of the sensor on the blade needs to be known in order to align the sensor coordinate system to the turbine coordinate system. For a sensor coordinate system as shown in Figure 3a and a mounting position of the sensor at $\gamma_{s}=0{}^{\circ}$ and $\beta_{s}=0{}^{\circ}$, the sensor coordinate system was then aligned to the turbine coordinate system at a rotation angle of $\alpha_{t}=90{}^{\circ}$ (horizontal alignment of the blade, with the blade pointing to the right) with $x_{s}=x_{t}$, $y_{s}=y_{t}$, $z_{s}=z_{t}$. It needs to be noted that the origin of the sensor coordinate system was located at the sensor, while the centre of the turbine coordinate system was located at the centre of the turbine hub. Therefore, the coordinate systems only differed in x-direction and rotation around the y-axis, and consequently, the subscripts for all axis and angles will be dropped in the following except for $\alpha_{t}$ and $\alpha_{s}$.

#### 2.1.1. Static Acceleration

The triaxial measurement of gravitational acceleration $a_{g}$ and centrifugal acceleration $a_{c}$ at a rotation angle $\alpha_{t}\left(k\right)$ are independent of the position and bending of the blade at rotation angle $\alpha_{t}\left(k-1\right)$ and will therefore be summarised as *static acceleration* $A_{s}$ the following. Static acceleration was simulated as

(1) $$A_{s}=R_{x}\;\cdot \;R_{z}\;\cdot \;\left(R_{y,t}\;\cdot \;\left(\begin{array}{c} a_{g}\\ 0\\ 0 \end{array}\right)+\left(\begin{array}{c} -a_{c}\\ 0\\ 0 \end{array}\right)\right),$$

with $R_{x}$ being the rotation matrix of the sensor around the x-axis due to pitch β, $R_{z}$ being the rotation matrix of the sensor around the z-axis due to the sensor orientation γ, and $R_{y,t}$ being the rotation matrix of the sensor around the y-axis of the turbine coordinate system due to rotational movement of the turbine. Acceleration due to gravity was adopted as $1g=9.81\;m/s^{2}$, while higher precision could be obtained when taking the place of manufacture for the accelerometer and the operating location of the turbine into account. Acceleration due to the centripetal force was calculated as $a_{c}={\left(2\pi f_{0}\right)}^{2}R$ for a rotation frequency $f_{0}$ of the turbine at a mounting radius *R* of the sensor.

**Figure 4:**
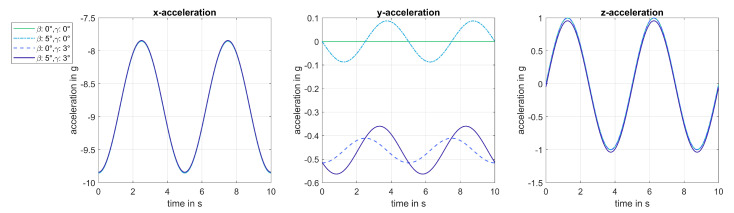
Simulation of the measured acceleration for varying mounting positions with β∈{0°,3°,5°}, γ∈{0°,3°,5°} and R=55 m: steady movement and no wind effects were simulated.

Depending on the orientation of the sensor, constant acceleration $a_{c}$ and acceleration due to gravity $a_{g}$ were measured by all three axes of an accelerometer to different extents (see Figure 4).

In case of constant bending of the blade, the effective position of the sensor in the coordinate system was changed correspondingly. In our approach, bending of the blade due to wind effects was simulated by applying the bending function $f_{blap/edge}=a_{flap/edge}\;\cdot \;x^{b}$ flapwise and edgewise to the blade.

*Flapwise bending* is caused by the normal forces acting on the blade and deflects the blade away from the wind out of the rotor plane. *Edgewise bending* acts across the chord-wise axis of the blade and is caused by the forces in the rotational direction of the blades, which deliver the shaft torque for turning the motor [23]. Additionally, *torsional bending*, i.e., twist of the blade, occurs [24], although it is expected to affect the blade less than flapwise and edgewise bending due to the higher torsional stiffness of the blade.

The material properties of the blade must be known for calculating the exact bending function. Since material properties and exact geometry are frequently unavailable from the manufacturer, we kept our method independent of those parameters. Therefore, our approach focused on the change of the effective sensor position and the resulting changes in pitch and rotation (see Figure 3b).

Bending of the blade results in a change of the effective position of the accelerometer, i.e., radius *R*, pitch angle β, and orientation γ. In edgewise bending, the blade is bent within the rotational plane, which changes the effective position of sensor by a rotation around the $y_{s}$-axis at $\alpha_{s}$. Measurements of both the gravitational and the centripetal force are influenced by this rotation. In contrast, the measurement of the centrifugal force $a_{c}$ is not influenced by the rotational position of the blade $\alpha_{t}$ due to the rotational movement of the blade. In addition to edgewise bending, flapwise bending deflects the blade out of the rotational plane, which corresponds to a rotation around the z-axis by $\gamma_{s}$ just as torsional bending leads to a rotation around the x-axis at $\beta_{s}$.

Consequently, our model was adapted to

(2) $$A_{s}=\underset{R_{x}}{\underset{︸}{R_{x,s}\;\cdot \;R_{x,t}}}\;\cdot \;\underset{R_{z}}{\underset{︸}{R_{z,s}\;\cdot \;R_{z,t}}}\;\cdot \;R_{y,s}\;\cdot \;\left(R_{y,t}\left(\begin{array}{c} a_{g}\\ 0\\ 0 \end{array}\right)+\left(\begin{array}{c} -a_{c}\\ 0\\ 0 \end{array}\right)\right)$$

with $R_{x}$ and $R_{z}$ describing the overall rotation of the sensor due to pitch and orientation, respectively; $R_{x,t}$ and $R_{z,t}$ describing the original rotation at the mounting position of the sensor due to pitch and orientation, respectively; and $R_{x,s}$ and $R_{z,s}$ describing the additional rotation for pitch and orientation due to bending, respectively.

#### 2.1.2. Dynamic Acceleration

Since the sensor coordinate system represents a moving coordinate system with reference to a fixed coordinate system, an accelerometer mounted on a turbine blade measures *Coriolis acceleration* and *Euler acceleration* in addition to static acceleration.

The acceleration due to the Coriolis force was calculated with

(3) $$A_{cp}=-2\;\cdot \;\vec{\omega }\times \vec{v_{r}}$$

as described in [25], with $\vec{\omega }$ being the rotational speed $\omega =2\pi f_{0}$ in the direction of the rotational axis $y_{t}$ and with $\vec{v_{r}}$ being the velocity of the movement from position $P_{k-1}$ to position $P_{k}$ in the rotating frame, i.e., the sensor coordinate system. The effective position of the sensor $P_{k}$ at rotation angle $\alpha_{k}$ with the same bending as at rotation angle $\alpha_{k-1}$ results in

(4) $$P_{k,b_{k}=b_{k-1}}=R_{yt}\left(\alpha_{t,k}-\alpha_{t,k-1}\right)\;\cdot \;P_{k-1}$$

and, consequently, the velocity results in

(5) $$v_{r}=\frac{d}{dt}\left(P_{k}-P_{k,b_{k}=b_{k-1}}\right).$$

In contrast to Coriolis acceleration, *Euler acceleration* relates to the reference frame, i.e., the turbine coordinate system, and results from a nonconstant angular velocity as [25]

(6) $$A_{e}=-\frac{d}{dt}\vec{\omega }\times P_{k}.$$

Since Coriolis acceleration relates to the sensor coordinate system, the resulting changes in position were small in comparison to Euler acceleration. Both accelerations will be referred to as *dynamic acceleration* $A_{d}=A_{cp}+A_{e}$ in the following.

#### 2.1.3. Overall Model

The overall acceleration measured by the sensor then resulted in a combination of both static and dynamic acceleration:

(7) $$A_{s,d}=\left[\begin{array}{c} a_{x}\\ a_{y}\\ a_{z} \end{array}\right]=A_{s}+A_{d}.$$

Dynamic acceleration only occurred if bending of the blade was not constant (Coriolis acceleration) or if the angular velocity was not constant (Euler acceleration). In the following, *tower shadow*, the most dynamic periodic wind effect, has been chosen for demonstration purposes.

**Figure 5:**
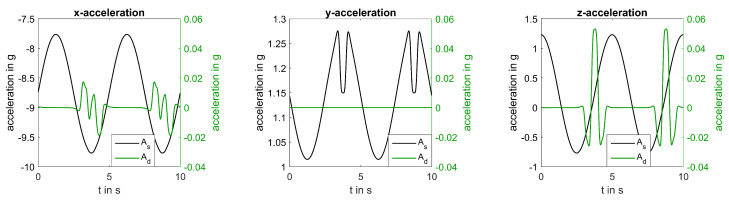
Comparison of static acceleration and overall acceleration of all three axes simulated for β=γ=0° and R=55 m.

Tower shadow specifies a decrease in the flow field near the tower ($\alpha_{t}=180{}^{\circ}$) and was simulated according to

(8) $$V_{tower}\left(y,x\right)=V_{0}a^{2}\frac{y^{2}-x^{2}}{{\left(x^{2}+y^{2}\right)}^{2}}$$

with $V_{0}$ being the static mean wind speed, *a* being the tower radius, and *x* and *y* being the longitudinal and the lateral distances from the blade to the tower mid-line, respectively [26]. Simulated 3D acceleration in the presence of tower shadow for a sensor mounted at a turbine blade can be seen in Figure 5. A characteristic change in static acceleration can be seen at a rotation angle of 180 when the blade passes the tower. Dynamic acceleration slightly increases before and after the blade passes the tower due to a change in angular velocity; however, overall acceleration is dominated by static acceleration.

### 2.2. Bending Shapes

Measured acceleration in alternate bending conditions was then analysed by using the model described in Section 2.1. Periodically occurring differences between the model and measured acceleration were used to create bending shapes reflecting these differences with reference to the rotation angle $\alpha_{t}$. Bending shapes were then used to extract significant bending patterns and were prepared for an analysis with regard to reference data. For demonstration, tower shadow was simulated following Equation (8).

#### 2.2.1. Estimation of the Rotation Angle

The rotation angle $\alpha_{t}$ had to be estimated to relate the temporal occurrence of the signal to the position of the sensor across the rotational circle of the blade.

First, the measurement axis with the most uniform acceleration was used to estimate the rotation frequency $f_{0}$. According to our model, the sensor axis $x_{s}$ was aligned best to the direction of the centripetal force. Therefore, the sensor axis $x_{s}$ was affected by rotational stiffening effects of the blade and experienced the least fluttering and noise.

The influence of noise and nonuniform rotation was minimised by applying a moving mean filter with a filter length of 10% of the signal length. Then, the rotation frequency $f_{0}$ was estimated by the robust nonlinear least square fitting the model $a_{x,fit}=m_{1}\;\cdot \;\left(2\pi f_{0}t+m_{2}\right)+m_{3}$ to measured acceleration $a_{x}$.

**Figure 6:**
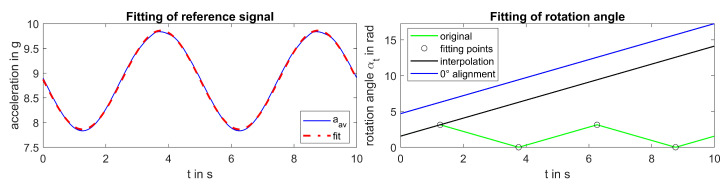
**Left**: fitting a reference signal to measured av-acceleration for angle estimation. **Right**: estimation of rotation angle $\alpha_{t}$ at constant angular speed.

Second, the vector sum $a_{av}=\sqrt{a_{x}^{2}+a_{y}^{2}+a_{z}^{2}}$ was used for calculating the rotation angle $\alpha_{t}$ since it represented maxima and minima of the 1g-modulation independently of pitch and orientation of the sensor. Again, robust nonlinear least square fitting was used to fit the model $a_{av,fit}=n_{1}\;\cdot \;sin\left(2\pi f_{0}t+n_{2}\right)+n_{3}$ to $a_{av}$, with $f_{0}$ being adopted from the first calculation step (see Figure 6, left). Then, the rotation angle was calculated as $\alpha_{t}=\cos{\left(a_{av,fit}\right)}^{-1}$. Since the reference signal had to reflect the constant angular speed of the turbine in case of uniform rotation, a straight line was fitted to all data points in which $\alpha_{i}-n\;\cdot \;\pi /2<\epsilon$ to obtain the rotation angle $\alpha_{t}$ (see Figure 6, right).

#### 2.2.2. Model Fitting

With $\alpha_{t}$ and $f_{0}$ known, the model developed in Equation (2) was then used to fit a reference signal $S_{x,y,z}$ describing uniform bending according to

(9) $$S_{x,y,z}=R_{x}\left(\beta \right)\;\cdot \;R_{z}\left(\gamma \right)\;\cdot \;R_{y,s}\left(\alpha_{s}\right)\;\cdot \;\left(R_{y,t}\left(\alpha_{t}\right)\;\cdot \;\left(\begin{array}{c} a_{g}\\ 0\\ 0 \end{array}\right)+\left(\begin{array}{c} -a_{c}\\ 0\\ 0 \end{array}\right)\right)$$

to measured acceleration $A_{x,y,z}$ describing nonuniform bending by minimising the cost function

(10) $$J=\left|\right|S_{x,y,z}-A_{x,y,z}{\left|\right|}_{2}^{2}.$$

**Figure 7:**
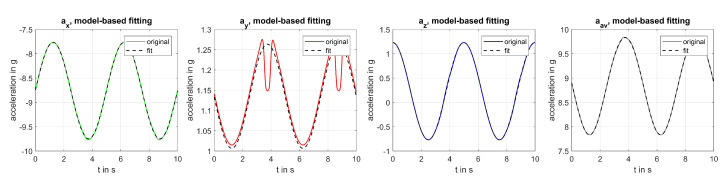
Model-based fitting for bending due to tower-shadow with sensor mounting position simulated as β=γ=0° and R=55 m.

Nonlinear least square fitting of parameters β, γ, $\alpha_{s}$, and *R* was used. While all angles were initialised at 0°, the initial estimate of the radius *R* was set at

(11) $$R_{0}=\frac{\sqrt{\sum_{i=x,y,z}\delta_{i}^{2}}}{{\left(2\pi f_{0}\right)}^{2}}$$

with $\delta_{i}$ being the DC acceleration $\delta_{i}=\min\left(a_{i}\right)+0.5\;\cdot \;\left(\max\left(a_{i}\right)-\min\left(a_{i}\right)\right)$ in each direction in $m/s^{2}$. Fitting boundaries were set at β=±45°, γ=±20°, $\alpha_{s}=\pm 30{}^{\circ}$, and $R=R_{0}\pm 3\;m$. Figure 7 shows the resulting model-based fitting for our test signal.

No fitting was applied to the vector sum $a_{av}$, and the resulting reference had been calculated from fitted functions in the x-, y-, and z-directions as

(12) $$S_{av}=\sqrt{S_{x}^{2}+S_{y}^{2}+S_{z}^{2}}.$$

The difference of model-based fitting and measured acceleration $D_{x,y,z,av}=S_{x,y,z,av}-A_{x,y,z,av}$ specifies the deviation of measured acceleration from uniform bending of the blade across the rotational movement of the turbine. Therefore, the deviation signal $D_{x,y,z,av}$ was used to analyse nonuniform bending in the following.

#### 2.2.3. Shape Computation

*Angular resampling* was used for analysing the difference signal $D_{x,y,z,av}$. The rotation angle $\alpha_{t}$ was used to align the signal to multiples of 0° to 360° rotations. Additionally, each rotation was resampled to $N_{s}=1000$ samples to enable comparability of different rotation frequencies.

For the following analysis, so-called *Bending Shapes* were created by taking the median of all single rotations for each measurement. Therefore, it could be assessed if differences from uniform bending occurred periodically and were significant or if they resulted from nonperiodic deviations due to noise or nonperiodic wind effects. The averaged cross-correlation coefficient of all rotations of $D_{x,y,z,av}$ in each direction was used to assess the significance of bending shapes. Only shapes with large cross correlation in all directions were analysed in the following since our approach focused on period alternate bending.

**Figure 8:**
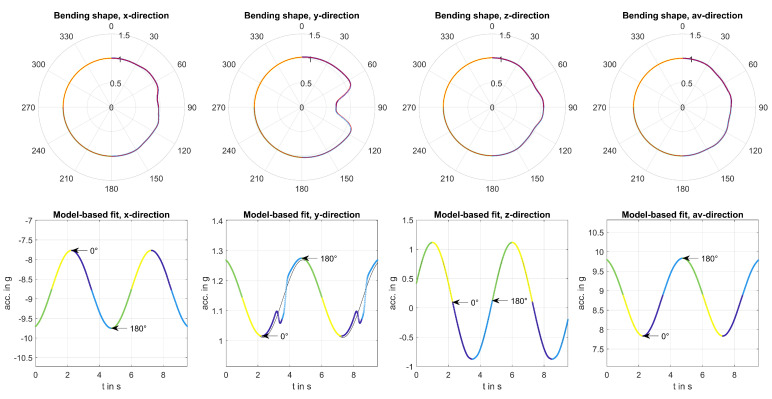
Bending shapes for simulated tower shadow for sensor mounting at β=0°=γ=0° and R=55 m at a rotation frequency of $f_{0}$ = 0.2 Hz.

Normalisation of bending shapes was applied for both pattern recognition and visualisation. Maximum of all deviations was used as a scaling factor. If shapes resulting from a single measurement were analysed, shapes were scaled to the maximum deviation across all directions. In case of an overall pattern recognition task, shapes were scaled separately to the maximum deviation for each direction. All shapes were then scaled to the deviation range of [0.5g,1.5g] for visualising shapes by means of polar plots. Resultingly, the unit circle represented uniform bending and radii smaller and greater than the unit circle corresponding to measured acceleration smaller and greater than the model-based reference, respectively. Figure 8 shows the resulting bending shapes for periodically varying bending due to tower shadow.

### 2.3. Pattern Recognition and Morphing Circle

The resulting bending shapes were analysed with regard to different turbine settings and bending effects. If external reference data are used in algorithm development, several difficulties can arise: First, reference data are frequently not available to the operator. Second, several reference measurements are not conducted on every turbine, e.g., measurements of the wind profile. Third, resolution of measurements might be insufficient, e.g., the pitch angle is often only measured with an accuracy of 1°. Therefore, we analysed bending shapes independently of reference data during the first stage of analysis.

**Figure 9:**
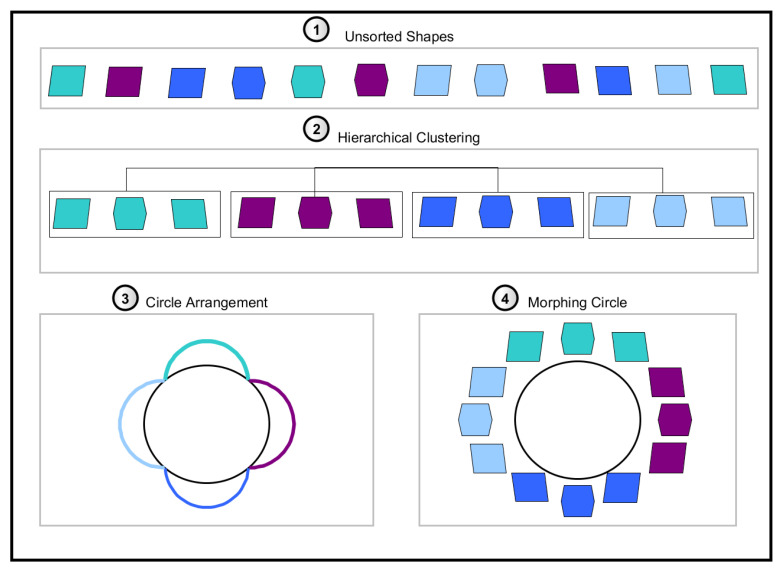
Overview of shape analysis and creation of bending patterns: colours of single elements represent different classes, and shapes of elements represent varying properties of bending shapes.

By analysing shapes regarding their similarity, pattern classes could be identified. This was a 4-step procedure which will be explained in the following and is illustrated in Figure 9.

**Figure 10:**
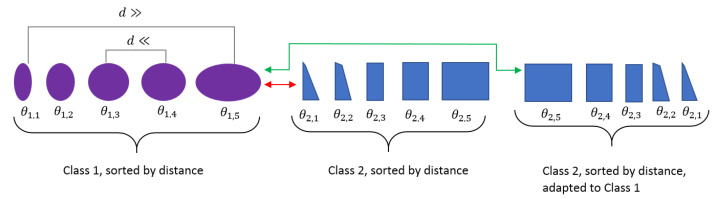
Sorting of shapes along a morphing circle. Left: arrangement of class elements $\theta_{i,k}$ by using Euclidean distances. Middle to right: reversing the order of elements to achieve matching boundaries between classes.

1. Unsorted Shapes:
  Identified bending shapes of varying environmental conditions (bending effects) and operational settings (mounting position of the sensor) were collected and normalised separately for each measurement direction as described in Section 2.2.3. Part 1 of Figure 9 displays the identified bending shapes as single elements, with colour representing affiliation to different classes.
2. Hierarchical Clustering:
  (a) *Hierarchical clustering* was used for assigning shapes to different classes by using a similarity measure [27]. The average Euclidean distance across all positions $\alpha_{t}$ between each pair of shapes was used as a distance measure. Agglomerate clustering was used, which uses bottom-up clustering by first treating all elements as single classes and then continuously merges classes. Part 2 of Figure 9 shows an example of assigning elements to four different classes.
  (b) The minimum number of elements per class was set to $N_{c}=3$ in order to exclude one-time events and to solely find classes representative of particular environmental or operational conditions. In case a class with a smaller number of elements was created, these elements were saved to a separate outlier class. We set the number of classes to N=10, which resulted in a total of 11 classes including the outlier class N+1. Iterative clustering was conducted as long as any class consisting of fewer elements than $N_{c}$ was created.
  (c) Finally, all elements of the outlier class were tested regarding their affiliation to any of the regular classes. For each class $C_{i}$, pair-wise Euclidean distances between all elements were used to form a distance group G1 and the distances from any element of the outlier class $C_{N+1}$ to all elements of class $C_{i}$ were used to form a distance group G2.
    Then, the significance of the affiliation of distances to groups G1 and G2 was tested by conducting an analysis of variances (ANOVA) with
    (13) $$F=\frac{S_{w}}{S_{b}},$$
    which tested the mean square (MS) $S_{w}$ within groups G1 and G2 against the mean square $S_{b}$ between both groups. The mean square was defined as the sum of squared deviations from the mean divided by the degrees of freedom [28]. In case elements from both groups stemmed from the same distribution, the F-score was small and the corresponding significance level *p* was large. The significance level was calculated for all classes $C_{i}$. If the highest significance level (largest *p*-value) exceeded p=0.1, the tested element of the outlier class was moved to the respective regular class $C_{i}$.
3. Circle Arrangement:
  The median of all class elements represented the bending pattern of each class. For visualisation, all patterns were arranged in a circle by minimising the Euclidean distances between patterns $P_{C_{i}}$
  (14) $$\min_{C_{i},C_{j}}\left|P_{C_{i}}-P_{C_{j}}\right|.$$
  Part 3 of Figure 9 displays a circle along which classes are arranged, with coloured bows representing the arrangement of classes along the circle.
4. Morphing Circle:
  Finally, all elements of each class were sorted by their Euclidean distances (see Figure 10). Elements were arranged within each class so that similar elements were located in the centre and the remaining elements were arranged to both sides with the first and last element having the largest distances between each other. This resulted in a morphing procedure from one bending shape to the other. At class boundaries, the order was either kept or reversed to align elements with the smallest distances, as shown in Figure 10. This arrangement could then be used for jointly visualising patterns and reference data. Part 4 of Figure 9 visualises the single elements along the morphing circle, with colour corresponding to different classes and shapes of single elements corresponding to varying properties of bending shapes.

## 3. Simulation

Alternate bending at varying mounting positions of the sensor has been simulated, and the resulting bending shapes and bending patterns have been analysed. First, the simulation of alternate bending is summarised in Section 3.1. Then, the results on bending shapes and patterns regarding different measurement axes as well as mounting radii are presented in Section 3.2 and Section 3.3.

### 3.1. Alternate Bending Effects

Alternate bending of the blade is caused by two different effects, which are (i) nonuniform wind conditions, i.e., yaw and wind shear, and (ii) gravity acting on the blade. As pointed out in detail in Section 2.1.3, blade bending consists of flapwise, edgewise, and torsional bending and can be measured by an accelerometer mounted at the blade tip.

The following effects have been simulated:

1. Wind shear: depending on the surface conditions on site, a nonuniform wind profile leads to an increase in wind speed with height; hence, the blade encounters different wind speeds across the rotation angle $\alpha_{t}$. Wind speed was simulated as $V\left(z\right)=V_{H}\;\cdot \;{\left(z/H\right)}^{\phi }$, with $V_{H}$ being the wind speed at hub height *H*, ϕ being the empirical wind shear exponent set to 0.2 in the simulation, and $z=h_{0}+R\;\cdot \;\cos\left(\alpha_{t}\right)$ being the effective blade height at rotation angle $\alpha_{t}$ and blade radius *R* [11].
2. Gravity: gravity counteracts bending due to wind load in case of a downwards movement of the blade and enhances bending due to wind load in case of an upwards movement of the blade. This leads to a sinusoidal increase and decrease of bending, which was simulated as $B\left(\alpha_{t}\right)=-c\;\cdot \;\sin\left(\alpha_{t}\right)$ for clockwise rotation with c=0.08.
3. Yaw: if the alignment of the turbine to the wind direction is not sufficiently exact, wind loads differ for the left and the right half-plane of the rotation circle. The yaw-afflicted wind profile was simulated following the definition in [9] as $V\left(\alpha_{t}\right)=V_{H}\;\cdot \;\cos\left(\theta_{m}\;\cdot \;\sin\left(\alpha_{t}\right)\right)$ for a yaw angle of $\theta_{m}=-20{}^{\circ}$.
4. Tower shadow: the simulation of tower shadow has already been discussed in detail in Section 2.1.3 and has been used as a test signal for visualising our method.

**Figure 11:**
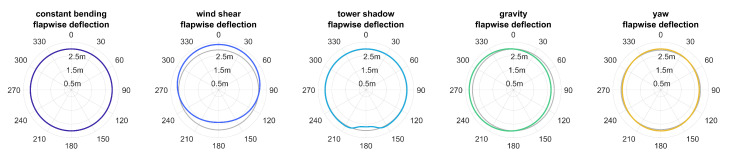
Flapwise deflection for simulated bending under bending effects at a mounting position of R=55 m on a 60 m blade.

The bending factor was then derived from the wind speed as $B\left(\alpha_{t}\right)=V\left(z\right)/V_{H}$. Flapwise and edgewise bending were simulated as

(15) $$f_{flap/edge}\left(\alpha_{t}\right)=B\left(\alpha_{t}\right)a_{flap/edge}\;\cdot \;L^{b}$$

with *L* being the length of the blade at the mounting position of the sensor. To the knowledge of the authors, there were no measurements on alternate bending available from the literature. Therefore, the bending simulation of a 30 m blade as described in [10] was used as an evidence. Bending was simulated for a 60 m blade as $a_{flap}=2\times 10^{-4}$, $a_{edge}=0.2\;\cdot \;a_{flap}$, and b=2.4. The ratio of flapwise bending to edgewise bending was slightly decreased in comparison to the referenced 30 m blade since a decrease in stiffness for longer blades was assumed. Figure 11 displays flapwise deflection for simulated alternate bending as defined in Equation (15).

### 3.2. Simulated Bending Patterns

**Figure 12:**
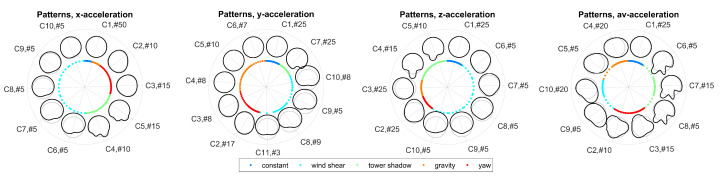
Effect of mounting angles on resulting bending patterns: mounting angles simulated as β and γ varying from −8 to 8 in 4 steps for a rotation frequency of $f_{0}=0.2$ Hz. Patterns are displayed along the morphing circle with Ci#j specifying the cluster number *i* with *j* members. Bending effects are displayed along the coloured inner circle.

Alternate bending was simulated for a 60 m rotor blade with sensors mounted at a blade radius of 55 m. First, the effect of the mounting angle has been evaluated. Figure 12 shows the resulting morphing circle for x-, y-, z-, and av-acceleration. One needs to note that clusters were not aligned across directions, i.e., cluster Ci of x-acceleration does not correspond to cluster Cj of y-acceleration.

The results show that bending shapes were assigned to different clusters for different bending effects with only one exception, which is cluster C1 in the x-direction representing both constant bending and bending due to gravity. In contrast, a bending effect might be represented by different classes since the number of clusters for hierarchical clustering was set to a constant number of 10 clusters. When comparing x-, y-, z-, and av-acceleration, the influence of the mounting angle of the sensor clearly vanishes in the av-direction. This is reflected by the fact that clusters were most similar for a distinct bending effect in the av-direction. Consequently, the effect of the mounting angle can be ignored when considering patterns of av-acceleration.

**Figure 13:**
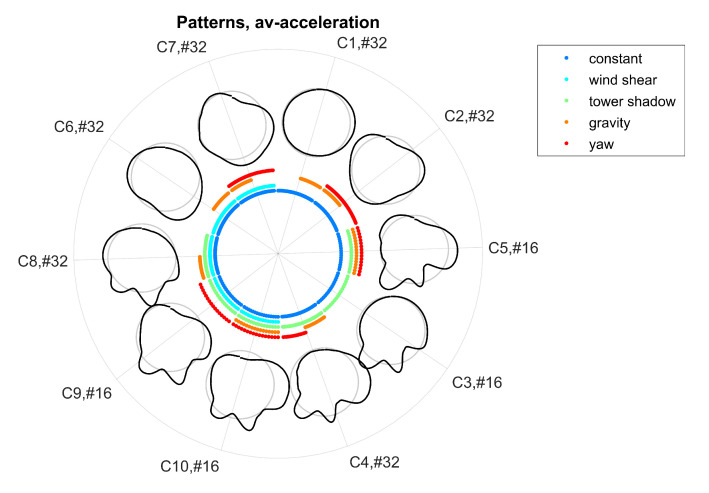
Resulting bending patterns for combined bending effects for a sensor mounting position of β=γ=0° and R=55 m and a rotation frequency of $f_{0}=0.2$ Hz: bending effects are displayed along the coloured inner circle.

The resulting morphing circle for all combinations of bending effects is displayed in Figure 13. The arrangement of classes along the morphing circle clearly represents different bending effects. For example, classes in the clockwise direction from C5 to C8 represent bending due to tower shadow, while classes in clockwise direction from C10 to C7 represent bending due to wind shear.

### 3.3. Resulting Bending Patterns

**Figure 14:**
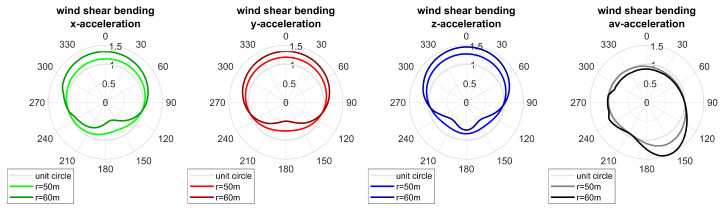
Bending shapes for sensors mounted at R=50 m, R=60 m, and β=γ=0°.

Additionally, the effect of the position of the sensor along the blade length was evaluated. Acceleration measurements were simulated for two sensors mounted at 50 m and 60 m. Figure 14 displays resulting bending shapes in the case of wind shear. The characteristics of shapes were more pronounced if the sensor was mounted at a larger radius of the blade, i.e., the deviation of shapes from the unit circle increased. This was to be expected since blade deflection increases with increasing radial position along the blade; therefore, variations in bending were reflected in the markedness of bending shapes. Consequently, the markedness of blade shapes can be used to assess the amount of alternate bending of the blade.

## 4. Real Data Experiment

Methods have been verified by applying model-based bending shapes to real data measured on the tip of wind turbine blades. The feasibility of shape computation as well as the generation of the morphing circle was tested, and the resulting bending shapes were evaluated with reference to simulated patterns.

### 4.1. Measurement Setup

Acceleration was measured by placing three sensors at a minimum of 80% blade length. To circumvent placement restrictions by support structures inside the blades, sensors were placed on the outside of the blade by integrating a triaxial Micro-Electromechanical Systems (MEMS) accelerometer (Analog Devices ADXL345 [29]) into a sensor solution developed by eologix [30]. This thin and robust sensor, originally developed for ice detection, is energy self-sufficient and powered by a solar cell. The sensors were mounted on the outside of the blades by means of self-adhesive erosion protection tape.

Acceleration was measured at 400 Hz in measurement campaigns of 10 s duration. Wireless data transfer in a licence-free short-range device frequency band was used to send data to a base station, which was mounted in the nacelle of the turbine and subsequently transferred data into a data base.

In total, three sensors were tested on two different blades. The first blade was 63 m long, and sensors S1 and S2 were placed at 88% and 98% of the blade length, respectively. Data were collected in a 2.5-month test period from mid-February till end of April. In this period, the blade temperature was between 1.7 °C and 22 °C.

The second blade was 49.5 m long, and the third sensor S3 was placed at 80% of the blade length. The test period was significantly longer, with 7 months of data collection from mid-December till mid-July. This also allowed for collecting data at a larger temperature range from −11.5 °C to 32.4 °C.

The two blades were located at two different sites with varying environmental conditions. A weather mast to collect more information about wind profiles, shear winds, etc. was not available. The blades were fully functional and both test turbines operated under standard conditions during the full test period.

### 4.2. Data Preprocessing

Preprocessing had to be applied to acceleration measurements before bending shapes were computed. First, nonidealities of the sensor were calibrated following the approach described in [31]. Calibration of measurements was performed during standstills of the turbine to remove the impact of constant offset and cross-axis sensitivity, which had been identified as the major nonidealities for a MEMS accelerometer as used in this study.

Second, mounting positions of the sensors were aligned to the model so that measurements in the x-, y-, and z-directions corresponded to measured directions $x_{s}$, $y_{s}$, and $z_{s}$ of the sensor coordinate system, as defined in Figure 3a.

Finally, only measurements taken during constant rotation frequency were considered in order to reduce nonstationary effects during gearing or pitching of the turbine. For this, only measurements with $0.01\;\mathrm{Hz}\leq f_{0}\leq 0.3$ Hz and minimum variability of the 1-g modulation amplitude were analysed.

In total, 569 measurements and 548 measurements were analysed during a 2.5-month test period for sensors S1 and S2, respectively, and 1749 measurements were analysed during a 7-month test period for sensor S3.

### 4.3. Results

Bending shapes were computed, and a morphing circle was created separately for all three sensors. Since reference measurements regarding blade bending are rarely performed by turbine operators and measurement resolution of operational data is often insufficient, no external reference data have been included into the analysis. Instead, the date and time of the measurement, the rotation frequency of the turbine, and the temperature measured by each sensor were used to analyse bending patterns and to prove the concept of our method.

#### 4.3.1. Cluster Size

**Figure 15:**
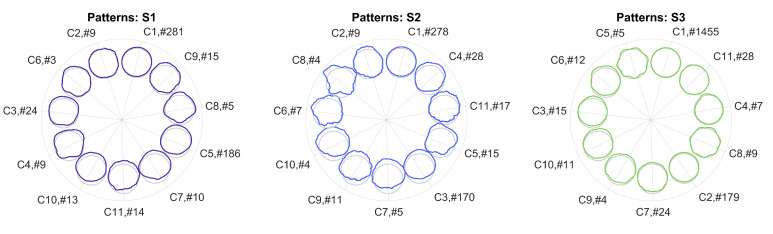
Morphing circle for sensors S1, S2, and S3 and patterns C1–C11 each in av-acceleration.

Bending patterns of the morphing circle are displayed in Figure 15 for all three sensors. For each sensor, more than 82% of bending shapes were distributed into two main classes; for sensor S3, class C1 itself represented 93% of shapes. The most frequent pattern, which was assigned to class C1 by the algorithm, was also the most regular one for all three sensors. This shows that uniform bending of the blade happens in many cases. However, bending patterns deviate from the unit circle for the second largest classes C5 and C3 for sensors S1 and S3, respectively, indicating nonuniform bending of the blade. When analysing the remaining patterns, distinct patterns are apparent which deviate from uniform bending, i.e., a unit circle shape, for all three sensors. The minimum number of elements per class was set to $N_{c}=3$; hence, each class does not correspond to a one-time event but represents certain operational and environmental conditions, e.g., pitch, yaw, and wind profile, which lead to alternate bending.

#### 4.3.2. Evaluation of Sensors S1 and S2

When comparing sensors S1 and S2 mounted on the same blade, similar patterns occur for both sensors. However, the analysis was performed separately for each sensor. Therefore, class numbers do not correspond to each other for different sensors. For example, class C5 of sensor S2 resembles class C8 of sensor S1 and class C8 of sensor S2 resembles class C4 of sensor S1 although the shape being more pronounced. Since sensor S2 was mounted at a larger blade radius than sensor S1, this comes as no surprise since the effect of alternate bending is expected to increase with increasing radius. A similar effect has also been observed in the simulation (see Figure 14).

**Figure 16:**
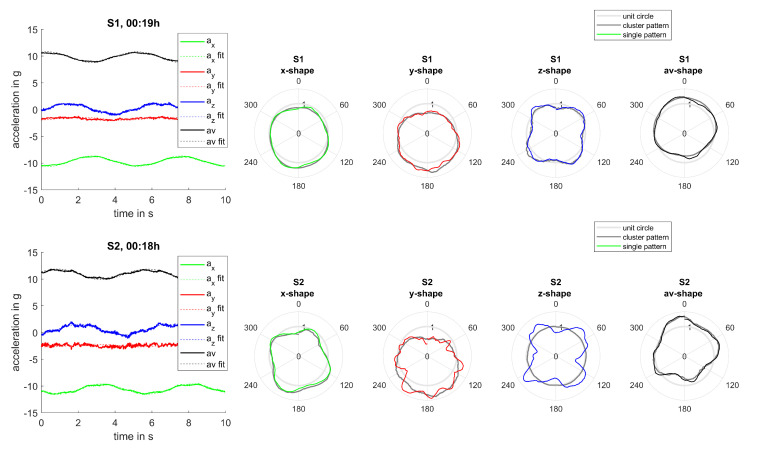
Joint analysis, event 1: Comparison of bending patterns for sensors S1 and S2.

**Figure 17:**
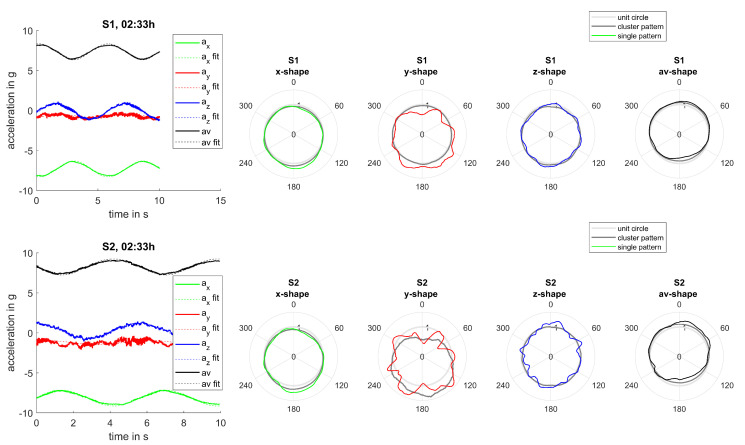
Joint analysis, event 2: Comparison of bending patterns for sensors S1 and S2.

Evaluating single measurements and the resulting bending patterns confirms those findings. Even though measurements were not scheduled synchronously for both sensors, events could be found for which measurements occurred within a 2-min interval and for which the rotation frequency varied less than 2%. Therefore, turbine settings and operational conditions can be assumed reasonably steady during those events. Two examples of such measurements can be seen in Figure 16 and Figure 17. Bending patterns clearly correlate for both sensors for all measurement directions and are more pronounced for sensor S2 mounted at a larger radius than sensor S1. Consequently, markedness of patterns can be used to assess the extent of alternate bending.

#### 4.3.3. Evaluation of Sensor S3

Bending patterns are expected to vary between blades due to deviating geometry and materials as well as between wind park sites due to varying wind profiles. Bending patterns significantly differed for sensor S3, which was mounted at both a different turbine type and at a different site. Generally, patterns were not as marked as for sensors S1 and S2; however, periodic deviations could be observed for patterns C5 and C8.

#### 4.3.4. Evaluation of Reference Data

**Figure 18:**
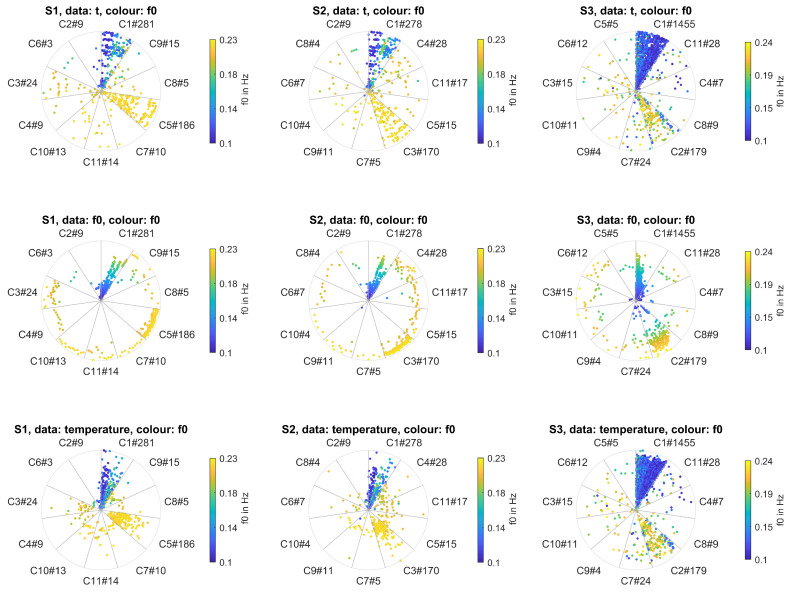
Visualisation of reference data for sensors S1, S2, and S3: normalised reference data are displayed across the radius of each polar plot. Top: measurement time *t* of a 2.5-month test period for sensors S1 and S2 and a 7-month test period for sensor S3. Middle: rotation frequency $f_{0}$ of 0.1 – 0.23 (S1 and S2) and 0.1 – 0.24 (S3). Bottom: Temperature ranges of 1.65 °C– 21.0 °C (S1), 1.9 °C– 21.7 °C (S2), and −11.5 °C– 32.4 °C (S3), respectively. The colour of data points corresponds to the rotation frequency of the turbine. Bending shapes of av-acceleration are displayed.

Date and time of the measurement, the rotation frequency, and the temperature of the blade were correlated with bending patterns. Reference data were normalised to [0,1] and visualised by displaying each reference measurement along the radius of the morphing circle, see Figure 18.

A clear relation between the rotation frequency and resulting bending patterns can be noted. For all sensors, low rotation frequencies resulted in more uniform bending and bending patterns were assigned to the most uniform class C1, while high rotation frequencies resulted in nonuniform bending corresponding to classes C5 (S1), C3 (S2), and C2 (S3). For sensor S3, there were few additional elements assigned to class C2 at low rotation frequencies since the two main shapes were very similar.

Generally, patterns were more variable and more pronounced for higher rotation frequencies despite the fact that blade stiffness increases with rotation frequency.

This may result from higher forces acting on the blade in wind shear and yaw conditions at higher wind speeds. At the same time, the results suggest that the effect of alternate bending due to gravity was low for the blades monitored in this study.

Additionally, certain bending patterns occurred at distinct rotation frequencies which can be observed for patterns C4, C3, and C6 (S1) and C6, C8, and C2 (S2). This effect had also been observed in [17]. Also, patterns C5 and C8 of sensor S3 occurred at a distinct rotation frequency at distributed measurement times. The corresponding patterns reflect periodic alternate bending at $f_{p}∼7f_{0}$, which suggests that blade modes were excited by the rotation frequency of the turbine.

Moreover, the temporal occurrence of patterns was analysed to detect changes in the bending behaviour of the blade over time. Short-term changes can be observed for class C6 of sensor S1, which occurs during a very limited time interval. The temporal occurrence can also be used to monitor a permanent long-term change in bending behaviour but could not be detected in this study due to a limited monitoring period.

An effect of the temperature on bending patterns could not be observed in the presence of the dominant influence of the rotation frequency.

Concludingly, a clear relation between bending patterns and the rotation frequency of the turbine could be found in the real data evaluation and proves the concept of the method.

#### 4.3.5. Evaluation of the Bending Simulation

When comparing real data bending patterns to simulated patterns as shown in Figure 13, it appears that tower shadow either hardly influenced monitored blades or that the simulation exaggerated the influence of tower shadow. However, influences of both simulated patterns C7 (apple-like patterns) and patterns C5,3,4,10, and 9 (tooth-like patterns) were found in real data patterns of sensors S1 and S2, e.g., patterns C8, C4, and C3 for sensor S1 and patterns C5, C6, and C8 for sensor S2. In our simulation, these patterns resulted from combining both vertically (wind shear) and horizontally (yaw) varying wind profiles, with tower shadow partly simulated (tooth-like patterns) and partly not simulated (apple-like patterns).

Therefore, the properties of simulated bending patterns are partly reflected in real measurement bending patterns. Even though it is not possible to relate the extent of each alternate bending effect to a real data bending shape, our method provides a strong indicator for detecting alternate bending.

## 5. Discussion

In this study, a method for monitoring alternate blade bending by placing an accelerometer at the blade tip was developed. Model-based bending shapes were derived by analysing the difference between measured and modelled acceleration at the blade tip. Hierarchical clustering was used to extract significant bending patterns. By introducing a minimum cluster size in the classification process, only representative patterns were identified and outliers due to nonstationary conditions were assigned to a separate outlier class. Additionally, bending shapes were arranged to a so-called morphing circle, in which patterns were sorted regarding their similarity. This method was then used to analyse bending shapes with reference to the rotation frequency, temperature, and temporal occurrence.

A simulation of alternate bending due to wind effects and gravity was successfully used to test our method regarding its distinguishability of alternate bending effects. Additionally, a real data experiment with three accelerometers revealed significant correlation of the rotation frequency with resulting bending shapes. While the impact of alternate bending was small for low frequencies, bending shapes were more pronounced and variable for high rotation frequencies in all sensors.

In future work, it is planned to collect data from (i) tip sensors on each turbine blade, (ii) sensors on several turbines of the same type, as well as (iii) sensors on different turbine types. Additionally, measurements will be synchronised to allow for a joint analysis of bending shapes. Finally, an evaluation of findings presented in this paper with reference to high accuracy wind profile measurements is highly desirable and will be conducted in the future.

## 6. Conclusions

The current trend of increasing the blade length for maximum energy capture results in larger loads and forces acting on the blades. Alternate blade bending in nonuniform wind profiles needs to be minimised to reduce loads and to prevent blade damage. Therefore, the lifetime of turbine blades is increased, costs are minimised, and the overall competitiveness of wind energy as a renewable source of energy is increased.

In this paper, we propose a novel approach for continuously monitoring blade bending in operation of the turbine, which is characterised by the following advantages:

- Accelerometers at the blade tip allow for a qualitative assessment of alternate bending at reasonable mounting effort.
- The sensors used in this study operate wirelessly and self-sufficiently; therefore, no restrictions on the mounting positions exist and sensors can even be used for retrofitting of existing turbines.
- No properties of the blade such as geometry and material, which are often not available by the operator, are needed.
- No environmental and operational parameters of the turbine are needed for evaluation. However, reference measurements at high accuracy are desirable for verification purposes.

Therefore, our method can be used to optimise turbine settings in nonuniform wind profiles with reasonable mounting effort and no restrictions on blade types. Additionally, our method can also be applied to detect deviating bending behaviour due to damage or aging of components in long-term monitoring applications.

## Abbreviations

The following abbreviations are used in this manuscript:

ICP	Individual pitch control
UWB	Ultra-Wideband
ANOVA	Analysis of variance
MS	Mean square
MEMS	Micro-Electromechanical Systems
IMU	Inertial measurement unit
